# A 13-year cohort study using clinical machine learning to differentiate bacterial and viral infections in young infants in a dengue hyperendemic region

**DOI:** 10.1093/tropej/fmag050

**Published:** 2026-07-22

**Authors:** Lucas J Cortés-Guzmán, Doris M Salgado, Carlos F Narváez

**Affiliations:** División de Inmunología, Programa de Medicina, Facultad de Ciencias de la Salud, Universidad Surcolombiana, Neiva, Huila, 41001, Colombia; Área de Pediatría, Departamento de Ciencias Clínicas, Facultad de Ciencias de la Salud, Universidad Surcolombiana, Hospital Universitario de Neiva, Neiva, Huila, 41001, Colombia; División de Inmunología, Programa de Medicina, Facultad de Ciencias de la Salud, Universidad Surcolombiana, Neiva, Huila, 41001, Colombia; Área de Pediatría, Departamento de Ciencias Clínicas, Facultad de Ciencias de la Salud, Universidad Surcolombiana, Hospital Universitario de Neiva, Neiva, Huila, 41001, Colombia

## Abstract

Differentiating bacterial from viral infections in febrile young infants is challenging, particularly in dengue-hyperendemic regions. We developed and internally validated a clinical machine-learning model to enhance diagnostic accuracy in this risk population in Colombia. We retrospectively analyzed a pediatric infectious admission cohort (<18 years) at a reference hospital in southern Colombia from 2007 to 2019. 4671 admissions (2251 bacterial and 2420 viral) were included. Nine clinical and laboratory variables were used to train an eXtreme Gradient Boosting (XGBoost) classifier. We divided the data into development (70%) and test (30%) sets, with Youden’s J statistics defining the optimal threshold. Penalized logistic regression (LR) and single-marker rules [leukocytosis, C-reactive protein (CRP)] served as comparators. The young-infant XGBoost achieved an area under the receiver-operating characteristic curve (AUC) of 0.896, outperforming LR (0.790) and single markers (0.746–0.706). Sensitivity was 93.5%, specificity 76.1%, positive predictive value 87.9%, and negative predictive value 86.4% in the temporal validation cohort. Discrimination was highest in children aged 6–10 years (AUC 0.967). CRP positivity, leukocytosis >16 × 10³ µl^−1^, and thrombocytopenia <150 × 10³ µl^−1^ were the most informative features. A nine-variable XGBoost model using routine clinical and hematologic variables accurately differentiated bacterial from viral infections in children from a low-resource dengue-endemic setting. Performance remained stable during temporal validation. Improved specificity with preserved sensitivity supports earlier targeted therapy and antibiotic stewardship. Multicenter studies and exploration of clinical challenges are the next steps for this kind of tool.

## Introduction

Acute infections remain the leading cause for pediatric hospital admission, accounting for about one-fifth of all febrile presentations worldwide [1]. Globally, there were 6.49 million deaths in children and adolescents younger than 20 years in 2021, with 78.5% occurring in children younger than 5 years [2].

Dengue is now endemic in 126 countries, primarily across tropical regions, with global cases increasing from 26.45 million in 1990 to 58.96 million by 2021 [3]. Rapid urbanization, inadequate water storage, and climate change have driven this rise, with South Asia and tropical Latin America most severely affected [3]. Colombia remains hyperendemic, with a major outbreak reported in 2024. Approximately 65% of municipalities provide ecological conditions that favor *Aedes aegypti* breeding and the continuous transmission of dengue [4]. By mid-August 2025, Colombia had reported 97 705 cumulative dengue cases, with the national incidence reaching 292.7 cases per 100 000 people at risk [5].

Distinguishing bacterial from viral illness in febrile children is notoriously difficult: 5%–15% of infants suffer serious bacterial infections, yet initial symptoms often overlap [6]. Isolated markers, such as leukocyte count or C-reactive protein (CRP), perform poorly with an area under the receiver-operating characteristic curve (AUC) of 0.58–0.60 because their distributions overlap widely across etiologies [7, 8]. A 2024 meta-analysis of 7755 infants showed that CRP ≥20 mg/L achieved only a partial AUC of 0.28, far below that of procalcitonin (0.72), and required thresholds <16 mg/L for meaningful rule-out [9]. Reliance on single tests therefore risks both missed bacterial disease and needless antibiotic use.

Improved diagnostic tools are urgently needed in pediatric settings, where dengue mimics bacterial sepsis. Machine-learning (ML) models can analyze big datasets to detect subtle multivariable patterns and generate faster, more accurate bedside predictions [10]. These approaches can improve the early differentiation between bacterial and viral infections, thereby optimizing overall management and antibiotic use [10]. Therefore, we developed and internally validated an ML model based solely on readily available clinical and hematologic variables to discriminate between bacterial and viral infections in children from a dengue-hyperendemic, resource-limited setting in southern Colombia.

## Materials and methods

### Study design, data source, and setting

We conducted a retrospective analytic cohort study at Hospital Universitario Hernando Moncaleano Perdomo, a referral healthcare center in southern Colombia. The hospital serves a mixed urban and peri-urban population, supporting generalizability to similar Latin-American centers but limiting external validity in rural or non-endemic regions. We analyzed a prospectively collected database of pediatric infectious disease admissions (under 18 years of age) in the infectious diseases unit and discharged between 1 January 2007 and 31 December 2019. Most laboratory and clinical data were recorded in qualitative or ordinal categories rather than raw numerical values.

### Eligibility criteria

Children under 18 years of age admitted with acute febrile illness (temperature ≥38°C or clinician-diagnosed fever requiring at least 24 h of hospitalization) were eligible. Exclusion criteria comprised underlying oncologic disease, postoperative care, non-infectious diagnoses, parasitic or fungal infections, incomplete records lacking essential identifiers or admission/discharge dates, and cases with more than 30% missing predictor variables.

### Outcome definition

The primary outcome was binary (bacterial = 1; viral = 0):

Bacterial infection (reference = 1): ICD-10 bacterial diagnosis plus either (i) microbiological confirmation (positive blood, urine, feces, respiratory culture, or rapid bacterial test) or (ii) prespecified clinical criteria and antibiotic therapy.

Viral infection (reference = 0): Laboratory-confirmed dengue (positive NS1 antigen and/or IgM-DENV), clinical diagnosis according to Pan American guidelines [11] or viral syndrome with negative bacterial cultures and no focal bacterial source. Coinfections (concurrent viral and bacterial diagnoses) were excluded to preserve a binary endpoint. Dengue and other viral infection subgroups were used as descriptive and analysis strata and were not used to develop the ML model.

### Predictor selection and coding

The overall model included CRP positivity, leukocytosis (>16 × 10³ µl^−1^), leukopenia (<5 × 10³ µl^−1^), normal platelets (> 150 × 10³ µl^−1^), and six age groups (<1 month, 1–12 months, 1–5 years, 6–10 years, 11–15 years, and >15 years). However, for descriptive analysis, the two oldest age groups were combined into a single category (>11 years; see Table 1) due to the small number of patients older than 15 years. Due to the diagnostic challenges in young infants [6], an extended infant-specific model was subsequently developed, incorporating nine variables selected based on clinical plausibility and odds ratio (OR) >1.5: age <1 year, leukocyte count (<5, 5–15, ≥16 × 10³ µl^−1^), platelets (<10, 10–49, 50–149, ≥150 × 10³ µl^−1^), hemoglobin (<9, 9–11.9, ≥12 g/dL), CRP (negative/positive), dyspnea, hepatomegaly, petechiae (absent/present), and admission temperature (continuous). Ordinal variables were integer coded; binary signs were coded as 0/1; and temperature was kept continuous.

**Table 1:** Demographic and clinical profile of pediatric infectious admissions: comparison of bacterial vs. viral infections and viral vs. coinfection groups.

Parameter	Bacterial *n* = 2251	Viral *n* = 2420	Coinfections *n* = 186	Bacterial vs. viral (OR, 95% CI)	*P-*value	Viral vs. Coinfections (OR, 95% CI)	*P-*value
**Sex, *n* (%)**							
Female	1,138 (50.6)	1,208 (49.9)	81 (43.5)	1.03 (0.92–1.16)	.614	1.30 (0.97–1.75)	.094
Male	1,102 (49.0)	1,207 (49.9)	105 (56.5)	0.97 (0.86–1.09)	.614	0.77 (0.57–1.03)	.104
**Age, *n* (%)**							
1–5 months	335 (14.9)	130 (5.4)	20 (10.8)	3.08 (2.50–3.80)	**<.001** *	0.47 (0.29–0.78)	**.005** *
6–12 months	495 (22.0)	311 (12.9)	32 (17.2)	1.91 (1.64–2.23)	**<.001** *	0.71 (0.48–1.06)	.092
1–5 years	951 (42.2)	942 (38.9)	96 (51.6)	1.15 (1.02–1.31)	**.023** *	0.60 (0.44–0.81)	**<.001** *
6–10 years	309 (13.7)	727 (30.0)	29 (15.6)	0.37 (0.32–0.43)	**<.001** *	2.32 (1.55–3.49)	**<.001** *
>11 years	161 (7.2)	304 (12.6)	9 (4.8)	0.52 (0.42–0.65)	**<.001** *	2.89 (1.43–5.58)	**<.001** *
**Admission temperature (°C)** ^a^	37.0 (34.0–40.8)	36.9 (35.0–40.7)	37.0 (35.0–40.5)	−	**<.001** *	−	.327
**Antibiotic use, *n* (%)**	2106 (93.6)	265 (11.0)	142 (76.3)	366.7 (263.3–510.7)	**<.001** *	0.031 (0.024–0.040)	**<.001** *
**Hospital stay (days)** ^b^	5.0 (1–97)	3.0 (1–48)	6.0 (1–46)	−	**<.001** *	−	**<.001** *

DWS, dengue with warning signs.

a Median (min–max).

b The *P* value corresponds to the Mann–Whitney *U* test. Categorical variables were analyzed using the Fisher exact test or Pearson χ^2^ test.

* Bold values denote statistical significance (*P* < .05).

### Missing data handling

Continuous variables were imputed using age-stratified medians; categorical variables were imputed with the most frequent age-specific category within the development dataset.

### Sample size and model complexity

The analytic dataset comprised 4671 admissions (2251 bacterial and 2420 viral). The number of infants (<1 year) was *n* = 1275, providing ≥20 events per model parameter, consistent with recommended standards for the stable development of clinical ML models [12].

### Model development

Data were split 70%/30% (stratified) into development and test sets. An eXtreme Gradient Boosting classifier XGBoost (version 2.1.1; xgboost developers), Python (version 3.10.12; Python Software Foundation, Wilmington, DE, USA; 100 trees, *learning_rate *= 0.10, *max_depth *= 3, *random_state *= 42) was fitted. The optimal threshold (τ) from the development receiver-operating characteristic (ROC) was 0.473. For patients >15 years of age, τ = 0.40 maintained ≥80% sensitivity.

For infants (<12 months), we retrained an XGBoost model with the nine-variable set and a 10-fold cross-validation within the training set (*eta (learning_rate)* = 0.05, *n_estimators *= 300, *max_depth *= 3, *subsample *= 0.80, *colsample_bytree *= 0.80, *scale_pos_weight = viral/bacterial ratio), objective = “binary: logistic”, random_state = 42).* Youden’s J was used to determine the optimal τ. Penalized logistic regression (LR) served as the comparator. The same approach was repeated in the 6–10-year old subset (*n* = 1038) to examine whether age-specific tuning benefited older children.

### Model evaluation

Performance was assessed by ROC-AUC, Brier score, and calibration curves (Supplementary Fig. S1). Sensitivity, specificity, positive predictive value (PPV), and negative predictive value (NPV) were reported with 95% confidence intervals (CIs); Shapley additive explanations analyses were used to illustrate variable contributions (Supplementary Fig. S2). The final model was applied to infants (<1 year, *n* = 1275) for external-like validation (Supplementary Fig. S3).

**Figure 1:**
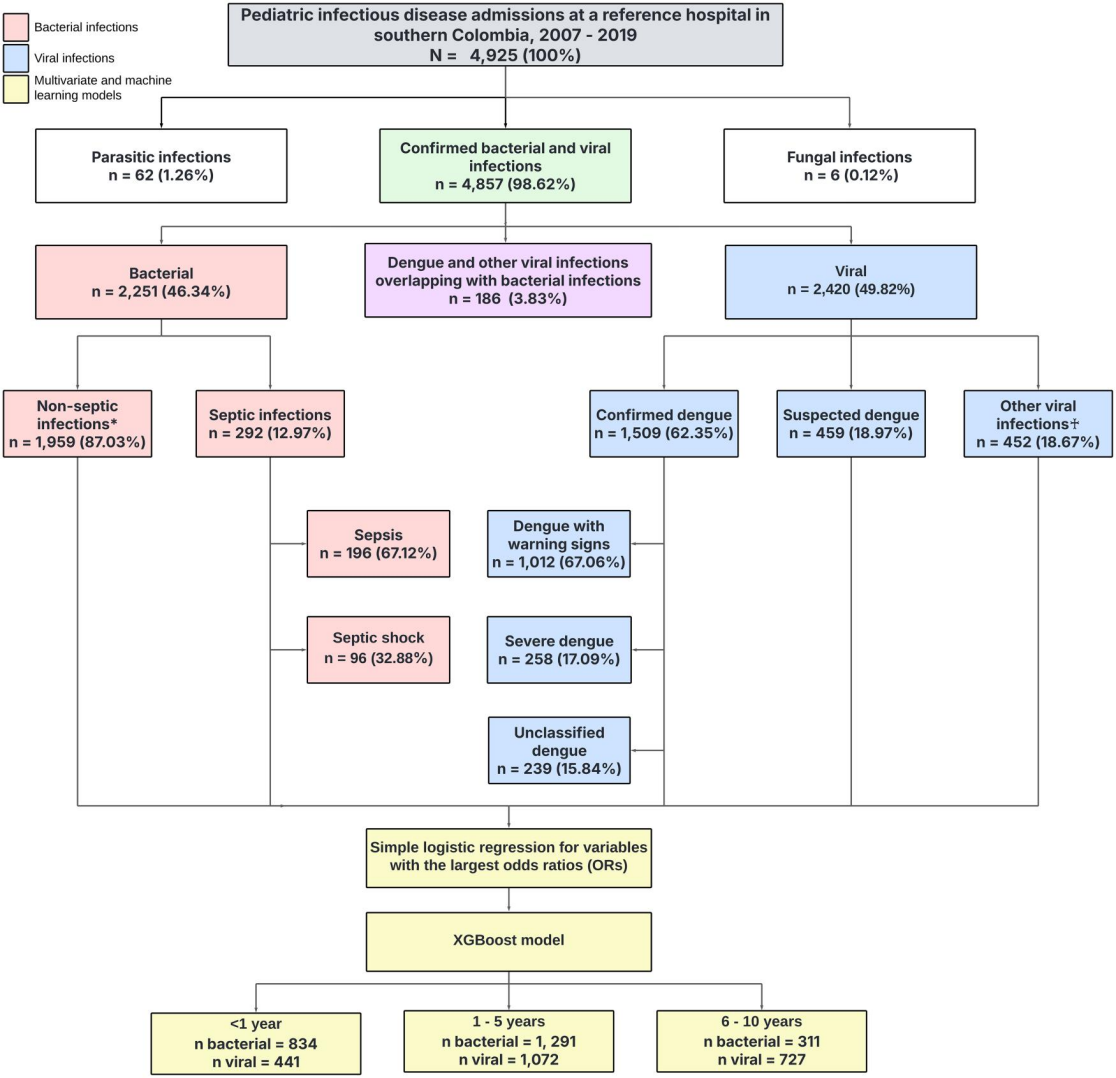
Study flow diagram illustrating patient selection and derivation of the bacterial, viral, and coinfection cohorts from pediatric infectious admissions (2007–2019). *Non-septic bacterial infections: urinary-tract infection (UTI), respiratory-tract infection (community-acquired pneumonia, bronchitis), acute gastroenteritis, skin-and-soft-tissue infection, osteo-articular infection, and other localized bacterial syndromes. †Other viral infections: influenza or influenza-like illness, acute viral gastroenteritis, adenovirus, common pediatric respiratory viruses, herpes viruses, and viral hepatitis.

**Figure 2:**
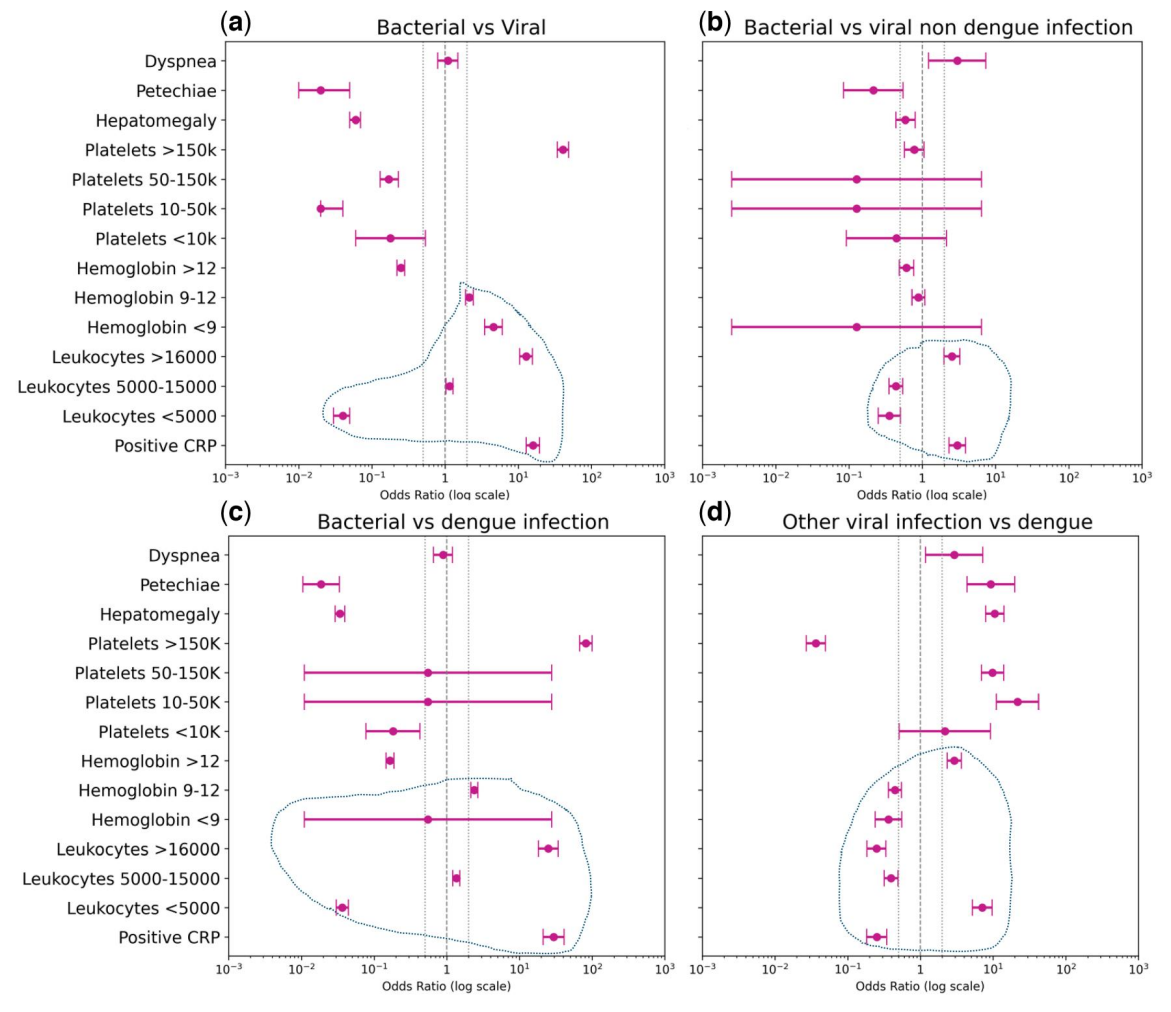
Forest plots show ORs and 95% CIs for clinical and laboratory variables across diagnostic comparisons. (a) Culture-confirmed bacterial infections vs. viral monoinfections. (b) Bacterial infections vs. non-dengue viral illnesses (e.g. influenza-like illness, viral diarrhea). (c) Bacterial infections vs. laboratory and clinically confirmed dengue (all severities). (d) Non-dengue viral infections vs. dengue infection.

**Figure 3:**
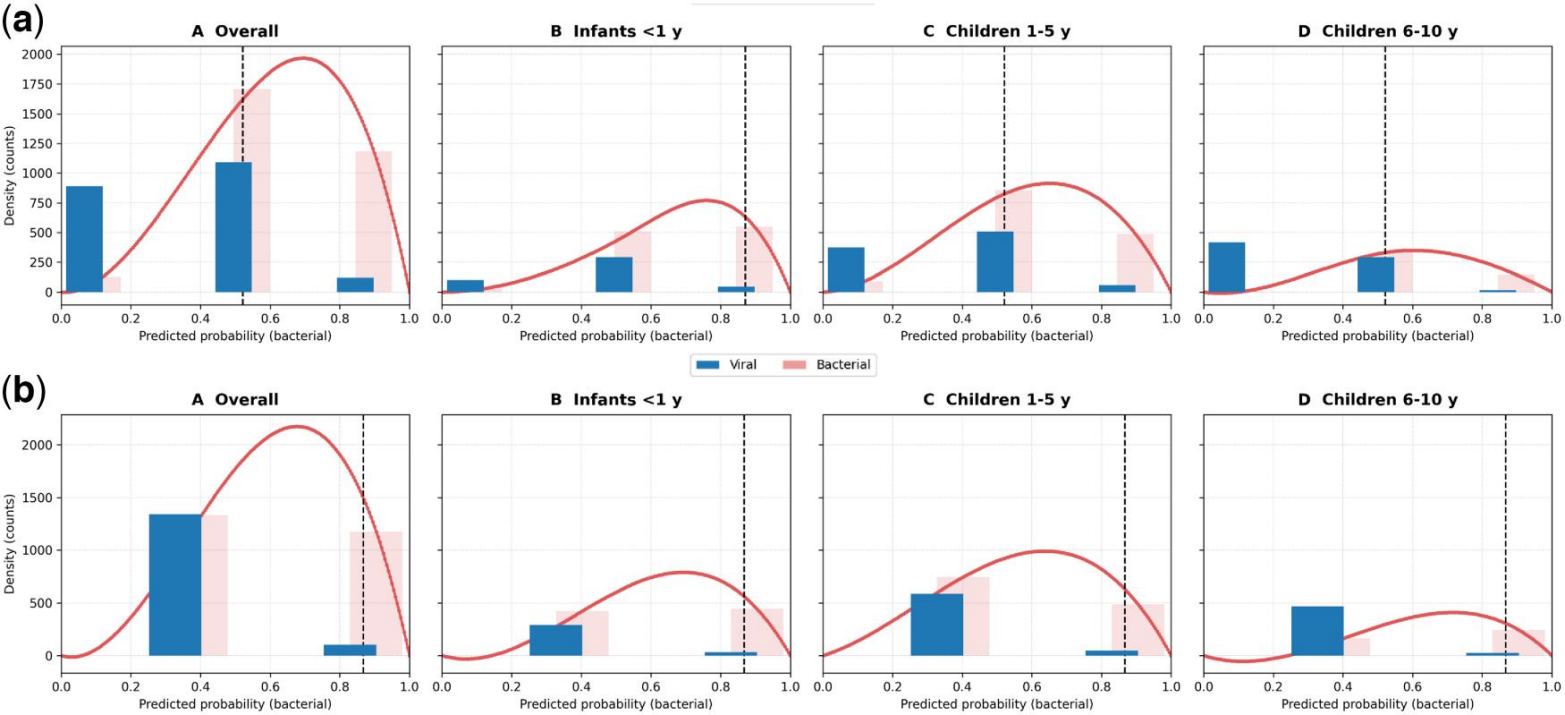
Distribution of model-predicted bacterial infection probability across age groups. Using leukocyte count (a) and C-reactive protein (b) as single predictors in the simple logistic regression models. Counts on the y-axis are normalized to equal total area within each diagnostic class, so bacterial and viral distributions are directly comparable across age strata.

For additional internal validation, we implemented a new model with temporal validation, excluding cases from the original test dataset to avoid model exposure. The data were randomly partitioned into training, testing, and temporal validation sets (70%/15%/15%) using identical hyperparameters. “External-like” validation used admissions from 1 January 2017 to 31 December 2019. Performance metrics from temporal and original test sets were compared to simulate real-world deployment during subsequent epidemic cycles.

### Statistical analysis

Categorical variables are presented as *n* (%) and continuous variables as median (range). Proportions were compared with χ^2^ or Fisher’s exact test and continuous variables with Mann–Whitney U test. ORs with 95% CIs accompany two-sided *P* value (significant *P* < .05). Analyses were conducted in Python (pandas 2.2, scikit-learn 1.5, statsmodels 0.15, matplotlib 3.9).

### Ethics approval

This study was conducted in accordance with the ethical principles of the Declaration of Helsinki and complied with all applicable ethical and regulatory requirements governing biomedical research involving human participants in Colombia. The study protocol was reviewed and approved by the Ethics, Bioethics, and Research Committee of Hospital Universitario de Neiva (Approval No. 008-006-2017).

## Results

### Characteristics of the cohort

Figure 1 summarizes the 4925 pediatric infectious disease admissions to the reference hospital, showing initial classification into parasitic, bacterial/viral, and fungal infections. Within the bacterial/viral group, 2251 admissions were bacterial and 2420 viral, while 186 bacterial–viral coinfections were excluded to retain a binary outcome. Bacterial cases were further divided into non-septic and septic presentations, and viral cases into confirmed dengue, suspected dengue, and other viral infections, with dengue stratified by clinical severity.

### Age distribution and clinical severity

Bacterial infections were concentrated in younger infants. Compared with viral infections, the odds of bacterial infection were higher among children aged 1–5 months (OR 3.08, 95% CI 2.50–3.80; *P* < .001) and 6–12 months (OR 1.91, 95% CI 1.64–2.23; *P* < .001). Bacterial admissions were also associated with longer hospitalizations (median 5 vs. 3 days; *P* < .001). Infants were overrepresented among sepsis cases, with ORs of 5.04 (95% CI 2.81–9.04) for 1–5 months and 5.56 (95% CI 3.80–8.16) for 6–12 months (both *P* < .001). Table 1 and Supplementary Table S1 show complete group counts and comparative data. Septic shock had the longest hospital stay (median 18 days), whereas severe dengue had a median stay of 4 days (Supplementary Table S2).

Thus, infants, particularly those under 1 year, had significantly higher odds of, and greater severity of, bacterial infections than viral infections, with longer hospital stays and greater treatment intensity.

### Single-variable markers favor bacterial infection but show limited precision

Higher leukocyte counts, normal platelet counts, and CRP positivity increased the odds of bacterial infection, while thrombocytopenia and leukopenia were more frequently observed in viral cases (Fig. 2). Despite these associations, distributions overlapped substantially. This overlap resulted in modest diagnostic accuracy, reflected by wide CIs and moderate performance metrics in both univariable analyses.

### Platelet count best distinguishes dengue from bacterial and other viral infections, while leukocytosis and a positive CRP indicate bacterial disease

In univariable contrasts (Fig. 2), bacterial episodes compared with all viral infections showed higher odds of leukocytosis (>16 × 10³/µl) and positive CRP, with lower odds of leukopenia (<5 × 10³/µl) and thrombocytopenia (Fig. 2a and b; most effects were moderate to large, ORs ≳2 or ≲0.5). The pattern strengthened when dengue was the comparator: severe thrombocytopenia (< 10 × 10³/µl) strongly favored dengue, whereas normal/high platelet levels (>150 × 10³/µl), leukocytosis, and positive CRP favored bacterial infection (Fig. 2c). Dengue was distinguished from other viral infections by a leftward shift in platelet counts and a greater frequency of petechiae and hepatomegaly. In contrast, other viral infections showed comparatively fewer thrombocytopenic and leukopenic presentations (Fig. 2d).

### Distinctive laboratory patterns in the etiologic groups

In hyperendemic dengue settings, viral-bacterial coinfections were characterized by severe thrombocytopenia (odds 15- to 20-fold greater than in isolated bacterial infections), highlighting it as a distinguishing laboratory feature of concurrent dengue and bacterial processes (blue circles, Supplementary Fig. S4). Positive CRP and leukocyte count ≥16 × 10³/µl were higher in the bacterial groups than in the overall coinfections group. Notably, coinfections were further distinguished by the frequent presence of other clinical signs—such as petechiae and hepatomegaly—that are consistent with dengue pathophysiological mechanisms but are seldom seen in classic bacterial presentations (Supplementary Fig. S4). These laboratory and clinical distinctions reinforce the importance of multivariable approaches for accurate classification in endemic contexts, as overlapping features among routinely measured markers can otherwise obscure coinfection or atypical disease.

**Figure 4:**
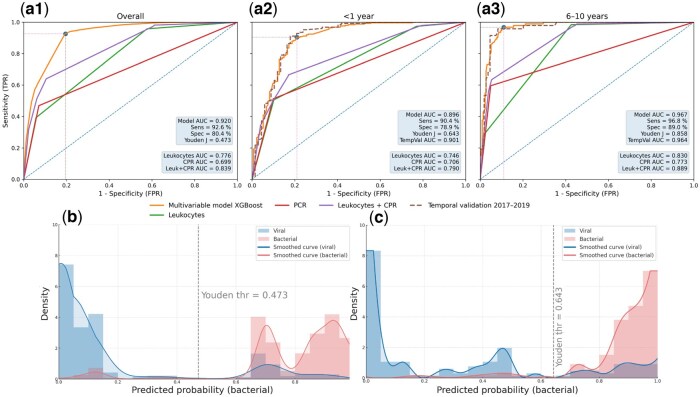
Age-stratified diagnostic performance of the nine-variable clinical XGBoost model for differentiating pediatric bacterial versus viral infections. (a1–a3) ROC curves compare the multivariable XGBoost model (orange) with single-marker rules based on total leukocyte count (green), C-reactive protein (CRP, red), and their logistic combination (purple). (b) Probability density profiles model-predicted probabilities for the whole cohort. Histograms are weighted by kernel density estimates (bandwidth = 0.05 for viral; bandwidth = 0.03 with reflection for bacterial) to emphasize distribution shape. (c) Equivalent probability density plot restricted to infants < 1 year of age.

### Single-marker rules (leukocytes or CRP) show limited discriminatory power across age groups

The limitations of single markers were evident in our cohort. Based on leukocyte count or CRP, these models showed substantial overlap in predicted probabilities for bacterial and viral infections across age groups. The density and histogram panels illustrate this limited separation in the overall cohort and in infants, children aged 1–5 years, and children aged 6–10 years (Fig. 3a for leukocytes and Fig. 3b for CRP). These results underscore the inadequacy of relying on isolated laboratory parameters and highlight the necessity of integrating automated, multi-variable assessments to achieve clinically meaningful accuracy in pediatric infectious disease diagnosis.

### Nine-variable clinical model outperforms single-marker approaches

Across all age strata, the multivariable model outperformed single markers and their simple LR (Fig. 4). In the overall 70/30 split, the model achieved an AUC of 0.920 (sensitivity = 92.6%, specificity = 80.4%), compared with 0.776 for leukocytes, 0.699 for CRP, and 0.839 for their simple logistic combination.

In infants aged ≤1 year (Fig. 4a2), XGBoost reached an AUC of 0.896 on the internal test set (sensitivity 90.4%, specificity 78.9%) and retained similar discrimination during temporal validation (2017–2019) with AUC 0.901 (sensitivity 93.5%, specificity 76.1%, PPV 87.9%, and NPV 86.4%). Performance remained well calibrated (Brier = 0.10), confirming minimal degradation when applied to later epidemic years.

Discrimination was highest in children aged 6–10 years (test AUC 0.967, sensitivity 96.8%, specificity 89%; temporal AUC 0.964, sensitivity 95.7%, specificity 87.2%, Fig. 4a3). Cross-validation within training folds yielded a mean AUC of 0.965 (SD 0.018) and Brier score of 0.064, underscoring internal stability. Overall, temporal validation mirrored the 70/30 performance (Fig. 4a2 and 4a^3^), demonstrating consistent model discrimination and calibration over time and supporting its robustness for prospective clinical deployment in endemic settings. Probability-density plots (Fig. 4b and c) demonstrated good separation of bacterial and viral predicted probabilities, with only modest overlap near the optimal decision threshold (Youden J index). This pattern was maintained in the infant subgroup (τ = 0.643) and school-age children (τ = 0.155), supporting consistent probability-based risk stratification.

In the held-out infant test set, classical bacterial syndromes (urinary tract infection, community-acquired pneumonia, sepsis) were labeled bacterial in ≥97% of cases. Dengue categories were mostly labeled viral (82–86% correct), with 14%–18% false-positive bacterial classifications reflecting overlapping inflammatory patterns (Supplementary Fig. S3). These results establish the advantage of using integrated, data-driven approaches for etiology classification in pediatric infectious disease, particularly in resource-limited and hyperendemic contexts.

## Discussion

Accurately distinguishing bacterial from viral infections, particularly dengue in young children, is complicated in hyperendemic settings due to early clinical overlap [13, 14]. In our 13-year cohort at a high-complexity hospital in southern Colombia, the distribution of infections and age profile closely matched those in prior studies of dengue-endemic regions [15]. Dengue was more prevalent in older children, paralleling endemic profiles from Latin America and Asia, co-circulating with other viruses (chikungunya and Zika) [16, 17] and, more recently, with SARS-CoV-2 [18].

Basic laboratory markers are commonly used, but they overlap across groups, limiting their discriminative power [7, 8]. Modest CRP elevations occur in viral and bacterial illnesses, reducing their standalone specificity [19, 20]. Atukuri et al. [21] noted that markedly high CRP levels were rare in dengue, suggesting an alternative diagnosis if disproportionately elevated. Our univariable analyses showed modest performance (leukocytes: AUC 0.776, CRP: AUC 0.699).

A large multicenter study across Asia and the Americas (IDAMS) (2023) confirmed the diagnostic importance of serial blood counts: dengue was characterized by falling white cell and platelet counts during days 2–5 of illness, whereas non-dengue fevers did not [15]. Hematologic and inflammatory markers are valuable as univariable clues; leukocytosis or an elevated CRP raises suspicion of bacterial infection, but none is sufficiently accurate [7, 8, 22]. Effective differentiation requires a combined multivariable approach.

Given the limitations of individual predictors, multivariable ML models offer superior discrimination by detecting complex nonlinear patterns [10, 23]. Our XGBoost classifier, trained on routine clinical data available in low-resource settings, outperformed LR (overall AUC 0.79) and single-marker approaches, particularly in infants, without sacrificing sensitivity.

Traditional scoring models incorporating multiple clinical and laboratory features have shown incremental benefits, with AUCs up to 0.990 and 0.981 [24], and systems combining up to 18 clinical and laboratory variables achieving sensitivities and specificities of 80%–90% for early dengue diagnosis [15]. A recent ML-based model for febrile infants (<90 days) achieved ∼0.99 AUC for predicting serious bacterial infection using numerous variables, including procalcitonin, electrolytes, and total protein, thereby vastly improving specificity over traditional criteria while maintaining 100% sensitivity [24], though it is unavailable in low-resource centers. Our model achieved comparable discrimination without the need for expensive biomarkers.

Early ML applications for dengue diagnosis using gradient-boosted approaches (XGBoost, LightGBM) reported improved diagnostic performance using accessible routine clinical and laboratory inputs [23, 25]. Our findings align with this trajectory: XGBoost captures nonlinear interactions and complex features that LR cannot capture. Temporal validation nearly matched internal test metrics: calibration remained stable, which is critical for endemic regions experiencing epidemic fluctuations.

A critical limitation of current algorithms is their static nature. Illnesses like dengue are dynamic (with defervescence and a platelet nadir around days 4–5), yet most guidelines do not fully account for laboratory trajectories over time [15]. The IDAMS study highlighted this gap, showing that dengue cases had declining white cell and platelet counts on days 2–5 of illness, whereas non-dengue fevers did not [15]. Future ML models might incorporate serial trends to markedly improve accuracy.

ML-driven clinical decision support integrates diverse inputs (symptoms, simple labs, epidemiologic context) into usable risk scores [26, 27]. Our study demonstrates that relatively basic variables used by ML achieve high discrimination; embedding them into electronic health systems or mobile apps could assist frontline providers with real-time decision-making. Prospective validation in target populations remains essential [13].

Accurate bacterial–viral differentiation has significant health-economic implications by promoting judicious antibiotic use and reducing costs associated with adverse events, antibiotic resistance, and readmissions [28]. ML algorithms like our XGBoost model enhance early targeted therapy, potentially decreasing inappropriate prescriptions and driving resistance [29]. Economic evaluations of antimicrobial stewardship interventions demonstrate substantial cost savings [30]. Embedding such models into clinical pathways could improve the efficiency of antibiotic stewardship programs, particularly in low-resource, dengue-endemic settings.

### Strengths and limitations

This study has several strengths. Our 13-year pediatric cohort from a dengue-hyperendemic region is among the largest reported cohorts of febrile young infants from such a region, which affects many worldwide; it also uses routinely available, low-cost clinical and laboratory variables, supporting its applicability in low-resource settings. In addition, temporal validation showed stable discrimination and calibration. Limitations should also be considered. The retrospective single-center design may introduce selection bias and limit generalizability. External multicenter validation was not available. Some variables were recorded categorically rather than continuously, and serial laboratory trends were unavailable. Prospective studies are needed to confirm clinical utility and impact on antibiotic use.

## Conclusion

A nine-variable XGBoost model using routine, simple clinical and hematologic variables accurately differentiated bacterial from viral infections in children in a low-resource, dengue-endemic setting. Performance remained stable during temporal validation. Multicenter studies and the exploration of clinical challenges are the next steps for this kind of tool.

## Supplementary Material

fmag050_Supplementary_Data

## Data Availability

All relevant data were included in the manuscript and the Supplementary material. Additional data will be obtained from the corresponding author.
