# Supplementary material for: A 13-year cohort study using clinical machine learning to differentiate bacterial and viral infections in young infants in a dengue hyperendemic region
Source: J Trop Pediatr. 2026 Jul 22;72(4):fmag050. doi: 10.1093/tropej/fmag050 (PMC13389304; doi:10.1093/tropej/fmag050)
Supplement: fmag050_Supplementary_Data [file fmag050_supplementary_data.docx]

**A 13-year cohort study using clinical machine learning to distinguish bacterial and viral infections in young infants in a dengue-hyperendemic region**

Lucas J. Cortés-Guzmán^1^, Doris M. Salgado^2^, Carlos F. Narváez^1,2^

1. División de Inmunología, Programa de Medicina, Facultad de Ciencias de la

Salud, Universidad Surcolombiana, Neiva, Huila, Colombia.

2. Área de Pediatría, Departamento de Ciencias Clínicas, Facultad de Ciencias de la Salud, Universidad Surcolombiana, Hospital Universitario de Neiva, Neiva, Huila, Colombia.

Corresponding author: Carlos F. Narváez MD., Ph.D. División de Inmunología, Programa de Medicina, Facultad de Ciencias de la Salud, Universidad Surcolombiana, Neiva, Huila Colombia. Facultad de Ciencias de la Salud, Universidad Surcolombiana, Calle 9 # 14 – 03, Barrio Altico, Neiva, Huila, Colombia.

e-mail: [cfnarvaez@usco.edu.co](mailto:cfnarvaez@usco.edu.co)

**Tables**

**Table S1. Demographic and clinical profile of pediatric infectious admissions.**

| **Parameter** | **Bacterial**  **n = 2,251** | **Viral**  **n = 2,420** | **Coinfections**  **n = 186** | **Bacterial sepsis n = 196** | **DWS n = 1,012** | **Septic shock**  **n = 96** | **Severe dengue**  **n = 258** | ***p-value*** |
| --- | --- | --- | --- | --- | --- | --- | --- | --- |
| Sex, n (%) |  |  |  |  |  |  |  |  |
| Female | 1,138 (50.6) | 1,208 (49.9) | 81 (43.5) | 110 (56.1) | 510 (50.4) | 41 (42.7) | 127 (49.2) | **0.002** |
| Male | 1,102 (49.0) | 1,207 (49.9) | 105 (56.5) | 86 (43.9) | 502 (49.6) | 55 (57.3) | 131 (50.8) | **0.007** |
| Age, n (%) |  |  |  |  |  |  |  |  |
| 1-5 months | 335 (14.9) ϫ | 130 (5.4) ϫ | 20 (10.8) | 23 (11.7) | 26 (2.6) | 18 (18.8) | 17 (6.6) | **<0.001** |
| 6-12 months | 495 (22.0) ϫ | 311 (12.9) ϫ | 32 (17.2) | 61 (31.1) | 76 (7.5) | 25 (26.0) | 40 (15.5) | **<0.001** |
| 1-5 years | 951 (42.2) | 942 (38.9) | 96 (51.6) | 81 (41.3) | 332 (32.8) | 33 (34.4) | 109 (42.2) | **<0.001** |
| 6-10 years | 309 (13.7) ϫ | 727 (30.0) ϫ | 29 (15.6) | 20 (10.2) | 394 (38.9) | 11 (11.5) | 69 (26.7) | **<0.001** |
| >11 years | 161 (7.2) | 304 (12.6) | 9 (4.8) | 11 (5.6) | 181 (17.9) | 9 (9.4) | 23 (8.9) | **<0.001** |
| Admission temperature °C† | 37.0 (34.0 – 40.8) | 36.9 (35.0 – 40.7) | 37.0 (35.0 – 40.5) | 38.0 (35.0 -40.8) | 36.7 (35.0 – 39.9) | 36.3 (35.2 -39.1) | 36.9 (35.3 – 39.5) | **<0.001** |
| Antibiotic use, n (%) | 2,106 (93.6) | 265 (11.0) | 142 (76.3) | 188 (95.9) | 47 (4.6) | 91 (94.8) | 61 (23.6) | **<0.001 ϴ** |
| Hospital stay (days)† | 5.0 (1 - 97) | 3.0 (1-48) | 6.0 (1 – 46) | 8 (1-77) | 3.0 (1 – 37) | 18.0 (2–77) | 4.0 (1 – 21) | **<0.001** |

† (median, min–max). DWS, dengue with warning signs. The p-value corresponds to the omnibus p-value. Statistical significance was reached (p < 0.05) ϴ Cramér’s V = 0.821. ϫPrimary differences are age differences in bacterial vs viral comparison: 1-5 months got an OR of 3.08 (2.5- 3.8) p <0.001, 6 -12 months OR: 1.91 (1.64 – 2.23) p<0.001, and 6– 10 years OR 0.37 p <0.001.

**Table S2. Demographic and clinical profile of pediatric infectious admissions comparison of severity groups bacterial versus viral infections.**

| **Parameter** | **Bacterial sepsis**  **n = 196** | **DWS**  **n = 1,012** | **Septic shock**  **n = 96** | **Severe dengue**  **n = 258** | **Bacterial sepsis vs DWS (OR, 95% CI)** | ***p-value*** | **Septic shock vs SD (OR, 95% CI)** | ***p-value*** |
| --- | --- | --- | --- | --- | --- | --- | --- | --- |
| Sex, n (%) |  |  |  |  |  |  |  |  |
| Female | 110 (56.1) | 510 (50.4) | 41 (42.7) | 127 (49.2) | 1.26 (0.93 – 1.71) | 0.160 | 0.77 (0.48 – 1.23) | 0.284 |
| Male | 86 (43.9) | 502 (49.6) | 55 (57.3) | 131 (50.8) | 0.79 (0.59 – 1.08) | 0.160 | 1.30 (0.81 – 2.09) | 0.284 |
| Age, n (%) |  |  |  |  |  |  |  |  |
| 1-5 months | 23 (11.7) | 26 (2.6) | 18 (18.8) | 17 (6.6) | 5.04 (2.81 – 9.04) | **<0.001** | 3.27 (1.61 – 6.66) | **0.001** |
| 6-12 months | 61 (31.1) | 76 (7.5) | 25 (26.0) | 40 (15.5) | 5.56 (3.80 – 8.16) | **<0.001** | 1.92 (1.09 – 3.38) | **0.023** |
| 1-5 years | 81 (41.3) | 332 (32.8) | 33 (34.4) | 109 (42.2) | 1.41 (1.02 – 1.94) | **0.035** | 0.70 (0.44 – 1.10) | 0.118 |
| 6-10 years | 20 (10.2) | 394 (38.9) | 11 (11.5) | 69 (26.7) | 0.18 (0.11 – 0.29) | **<0.001** | 0.36 (0.17 – 0.73) | **0.005** |
| >11 years | 11 (5.6) | 181 (17.9) | 9 (9.4) | 23 (8.9) | 0.27 (0.14 – 0.50) | **<0.001** | 1.09 (0.45 – 2.60) | 0.838 |
| Admission temperature °C† | 38.0 (35.0–40.8) | 36.7 (35.0 – 39.9) | 36.3 (35.2 -39.1) | 36.9 (35.3 – 39.5) | - | **<0.001** | - | 0.995 |
| Antibiotic use, n (%) | 188 (95.9) | 47 (4.64) | 91 (94.8) | 61 (23.6) | 482.50 (224.36 – 1,037.65) | **<0.001** | 58.78 (22.85 – 151.22) | **<0.001** |
| Hospital stay (days)† | 8.0 (1 – 77) | 3.0 (1 – 37) | 18.0 (2–77) | 4.0 (1 – 21) | - | **<0.001** | - | **<0.001** |

† (median, min–max). DWS, dengue with warning signs. SD, severe dengue. †The p-value corresponds to Mann-Whitney U, the other variables were used Fisher exact test or Pearson χ². Statistical significance was reached (p < 0.05).

**Supplemental Figures**


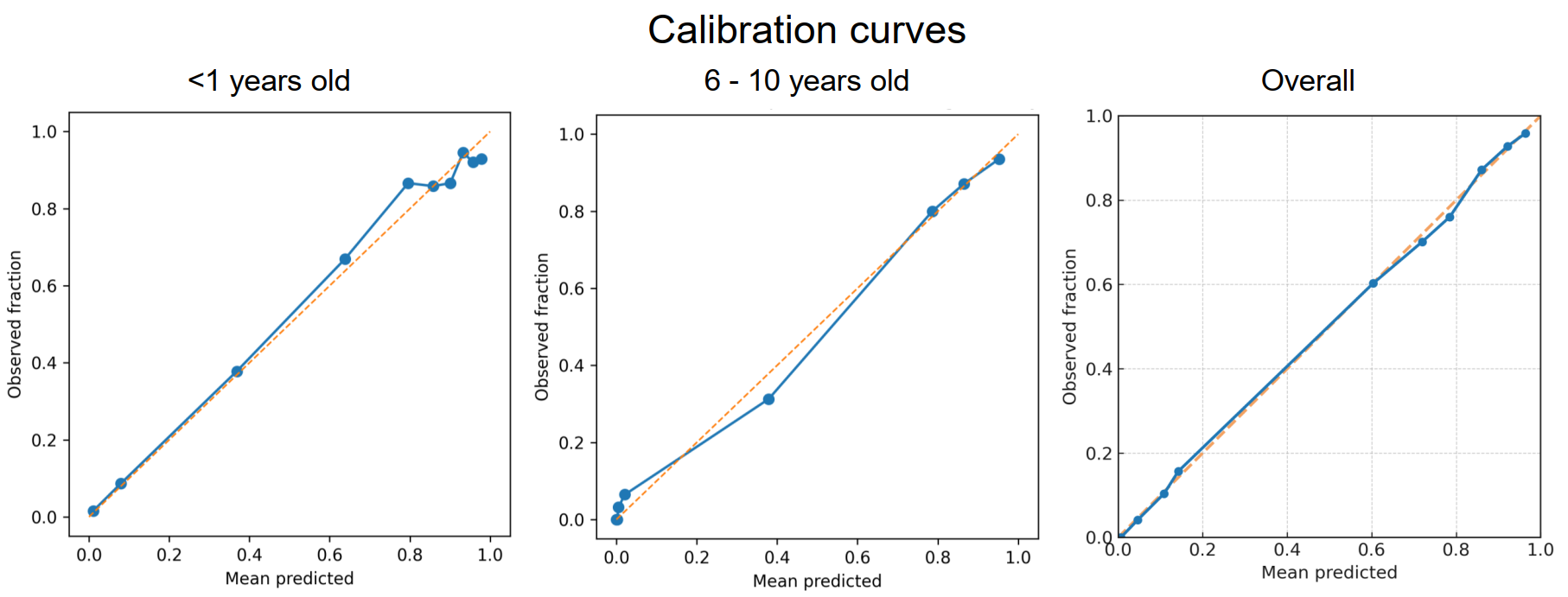
 **Supplemental Figure 1. Calibration curves for the XGBoost models are split by age group and the overall cohort.** The blue line represents the observed event rate within bins of predicted probability, whereas the orange dashed line indicates perfect calibration (observed = predicted). Closer agreement between both curves indicates better calibration and more reliable predicted probabilities.


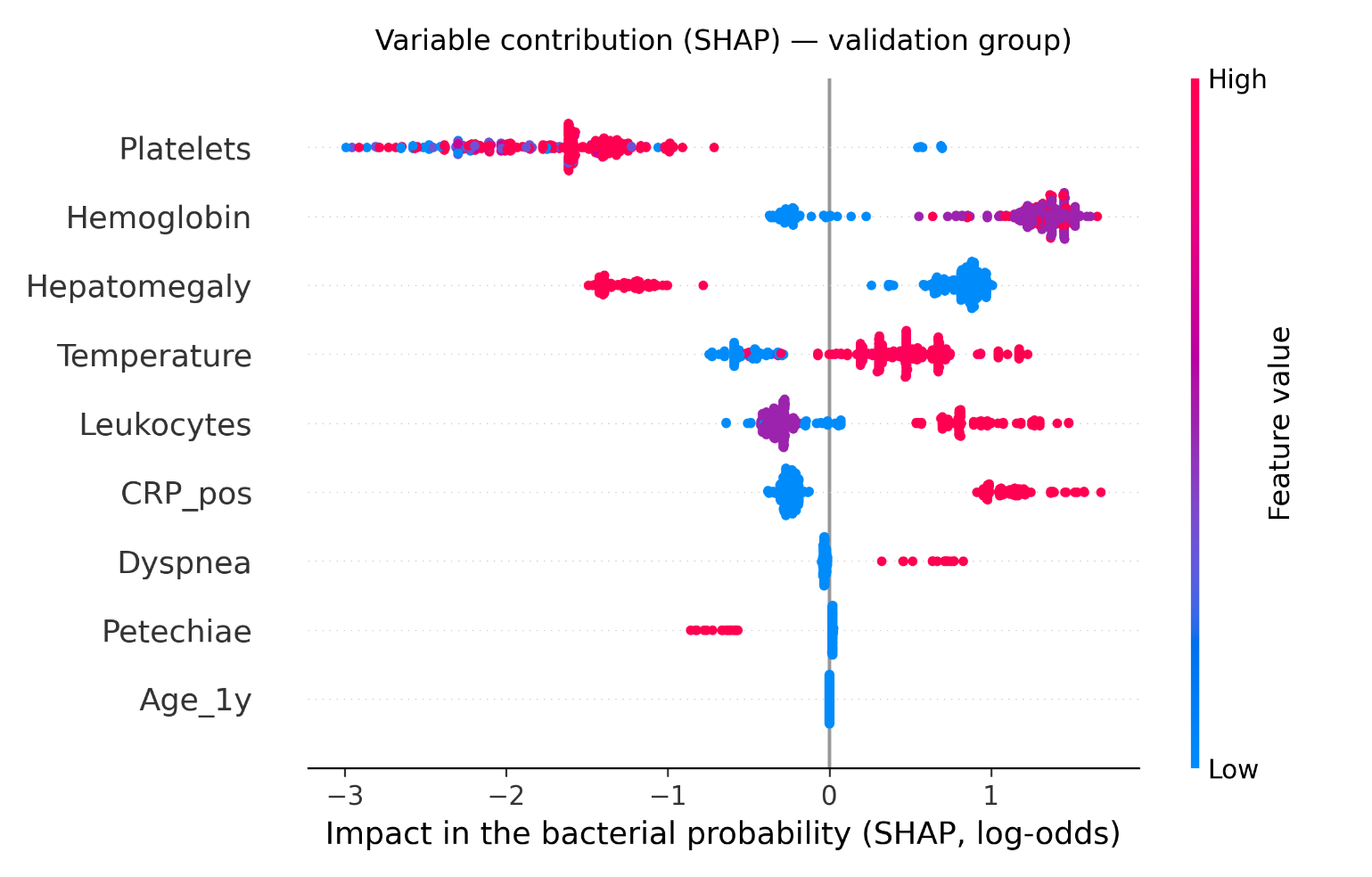


**Supplemental Figure 2. SHAP summary plot (validation cohort) for the 9-variable XGBoost model discriminating bacterial vs. viral infections.** Each dot represents one patient in the validation set. Dot color represents the feature value (blue = low, red = high). The vertical grey line marks no effect (SHAP = 0). CRP_pos = C-reactive protein positive; Age_1y = age <1 year.


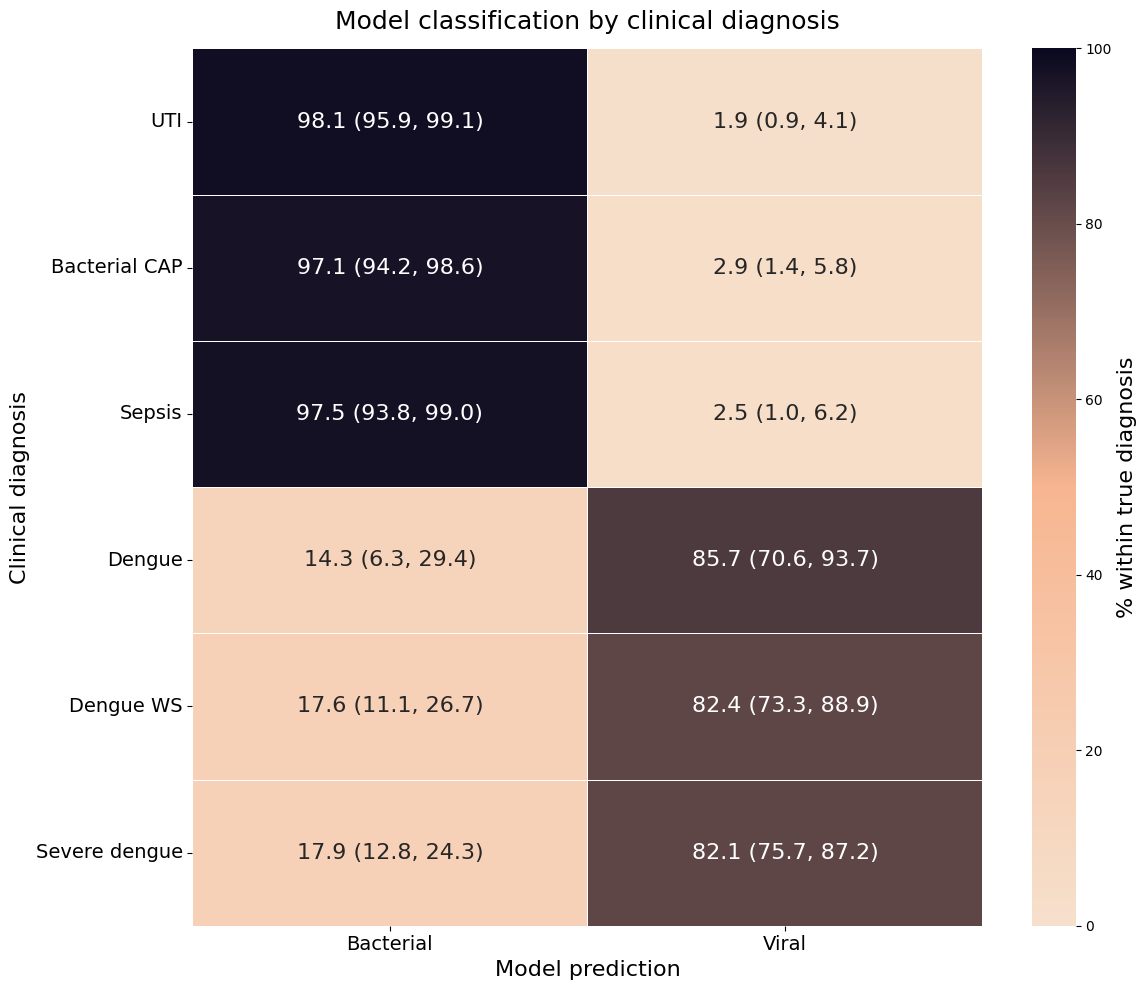


**Supplemental Figure 3. Heatmap of XGBoost model classifications across reference clinical diagnoses.** The 9-variable XGBoost classifier was applied to the held-out internal infant test set (n = 1,275). Rows represent the reference clinical diagnoses: urinary tract infection (UTI), community-acquired pneumonia (CAP), sepsis, non-severe dengue, dengue with warning signs (Dengue WS), and severe dengue, while columns show the label assigned by the model (bacterial vs. viral).


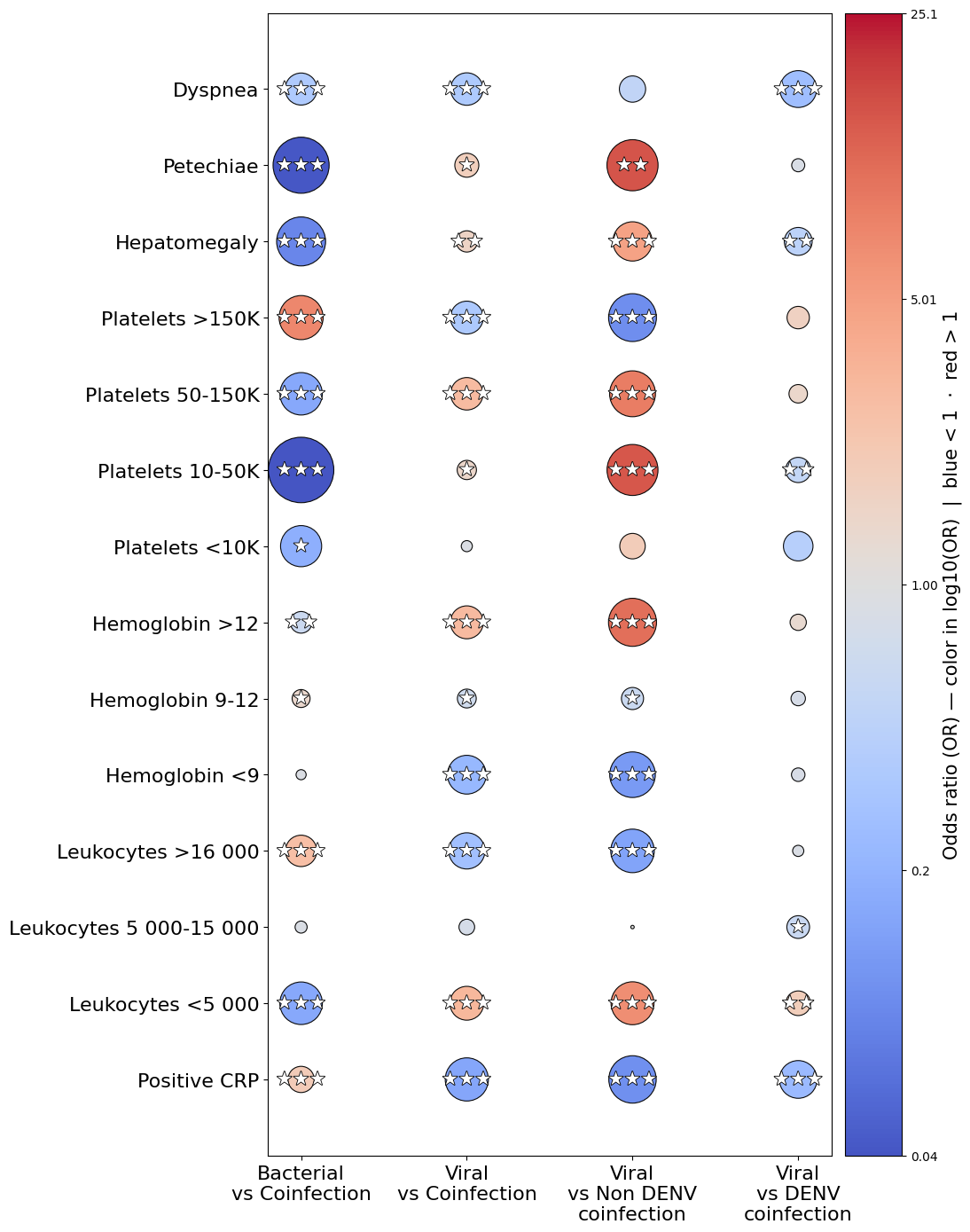


**Supplemental Figure 4. Bubble plot of clinical variables across four overlapping viral–bacterial infections with contrast odds ratios oriented toward the single pathogen reference group.** Each circle represents the odds ratio (OR) for the variable in that contrast: Bubble size is proportional to |log10 OR|, visually emphasizing stronger associations; asterisks indicate statistical significance from Wald tests: ★ p < 0.05, ★★ p < 0.01, ★★★ p < 0.001. The accompanying color bar contextualizes the direction and magnitude of the effect (deep red ≈ log10 OR ≈ +2.3 → OR ≈ 200; deep blue ≈ −2.3 → OR ≈ 0.005).
